# 3-Methyl­anilinium nitrate

**DOI:** 10.1107/S1600536810020738

**Published:** 2010-06-16

**Authors:** Melanie Rademeyer, David C. Liles

**Affiliations:** a Department of Chemistry, University of Pretoria, Pretoria 0002, South Africa

## Abstract

In the title compound, C_7_H_10_N^+^·NO_3_^−^, the 3-methyl­anilinium cations inter­act with the nitrate anions through strong bifurcated N^+^—H⋯(O,O) hydrogen bonds, forming a two-dimensional hydrogen-bonded network.

## Related literature

For related structures, see: Benali-Cherif *et al.* (2007 ▶, 2009 ▶). For hydrogen-bond motifs, see: Bernstein *et al.* (1995 ▶).
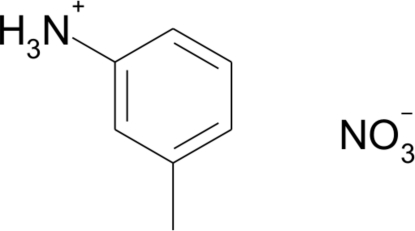


## Experimental

### 

#### Crystal data

C_7_H_10_N^+^·NO_3_^−^
*M* *_r_* = 170.17
Orthorhombic, 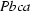

*a* = 10.6599 (14) Å
*b* = 9.7800 (13) Å
*c* = 16.401 (2) Å
*V* = 1709.9 (4) Å^3^
*Z* = 8
Mo *K*α radiation
μ = 0.11 mm^−1^
*T* = 293 K
0.40 × 0.32 × 0.05 mm

#### Data collection

Bruker (Siemens) P4 diffractometer
Absorption correction: multi-scan (*SADABS*; Bruker, 2001 ▶) *T*_min_ = 0.968, *T*_max_ = 0.988
8520 measured reflections
1659 independent reflections
1211 reflections with *I* > 2σ(*I*)
*R*_int_ = 0.032

#### Refinement

*R*[*F* ^2^ > 2σ(*F* ^2^)] = 0.040
*wR*(*F* ^2^) = 0.135
*S* = 1.06
1659 reflections
111 parameters
H-atom parameters constrained
Δρ_max_ = 0.17 e Å^−3^
Δρ_min_ = −0.14 e Å^−3^

### 

Data collection: *SMART* (Bruker, 2001 ▶); cell refinement: *SAINT* (Bruker, 2001 ▶); data reduction: *SAINT*; program(s) used to solve structure: *SHELXS97* (Sheldrick, 2008 ▶); program(s) used to refine structure: *SHELXL97* (Sheldrick, 2008 ▶); molecular graphics: *Mercury* (Macrae *et al.*, 2006 ▶); software used to prepare material for publication: *PLATON* (Spek, 2009 ▶) and *WinGX* (Farrugia, 1999 ▶).

## Supplementary Material

Crystal structure: contains datablocks global, I. DOI: 10.1107/S1600536810020738/kj2146sup1.cif

Structure factors: contains datablocks I. DOI: 10.1107/S1600536810020738/kj2146Isup2.hkl

Additional supplementary materials:  crystallographic information; 3D view; checkCIF report

## Figures and Tables

**Table 1:** Hydrogen-bond geometry (Å, °)

*D*—H⋯*A*	*D*—H	H⋯*A*	*D*⋯*A*	*D*—H⋯*A*
N1—H1*A*⋯O1^i^	0.89	2.05	2.943 (2)	178
N1—H1*A*⋯O2^i^	0.89	2.51	3.130 (2)	127
N1—H1*B*⋯O3^ii^	0.89	2.15	3.0221 (19)	167
N1—H1*B*⋯O2^ii^	0.89	2.37	3.078 (2)	136
N1—H1*C*⋯O3^iii^	0.89	2.01	2.879 (2)	166
N1—H1*C*⋯O1^iii^	0.89	2.47	3.176 (2)	137

Symmetry codes: (i) 
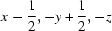
; (ii) 
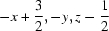
; (iii) 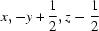.

**Fig. 1.**
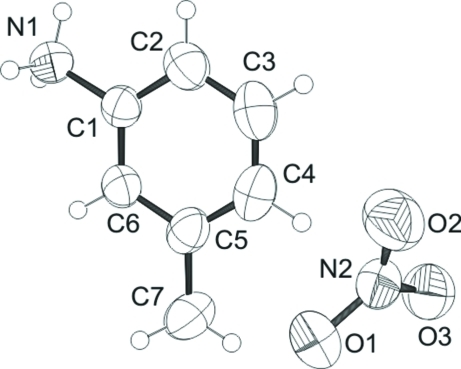
The asymmetric unit of the title compound showing the atomic numbering scheme. Displacement ellipsoids are shown at the 50% probability level.

**Fig. 2.**
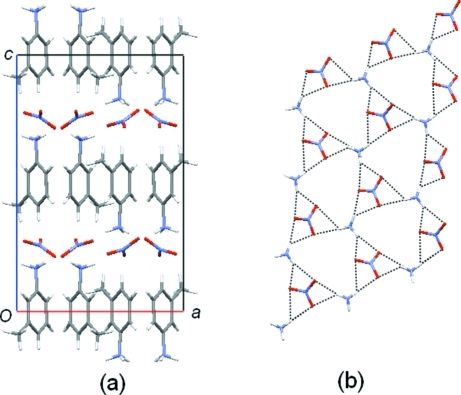
(a) Packing diagram of the title compound viewed down the b-axis.(b) N—H···O hydrogen bonding network in the title compound (dashed lines indicate hydrogen bonds).

## supplementary crystallographic information

### Comment

A fundamental understanding of the role of oxyanion geometry on molecular packing and non-covalent interactions in salt crystal structures is central to the fields of both molecular recognition and crystal engineering. The crystal structure of the title compound was determined as part of a project focusing on the role of anions when combined with alkylammonium or arylammonium cations. The structures of the related compounds *p*-toluidinium nitrate (Benali-Cherif *et al.*, 2009) and *o*-toluidinium nitrate (Benali-Cherif *et al.*, 2007) have been reported in the literature.

The molecular geometry and labelling scheme of the title compound is illustrated in Fig. 1. The asymmetric unit contains one 3-methylanilinium cation and one trigonal planar nitrate anion. A layered structure consisting of alternating organic and inorganic layers is exhibited by the title compound. The organic layers contain the hydrophobic part of the cation, while the inorganic layers comprise the ammonium groups and nitrate anions. The molecular packing of the title compound, viewed down the *b*-axis, is illustrated in Fig 2(a). In the organic layer pairs of cations alternate in orientation, with all the aromatic groups packing in a single row. Aromatic interactions are present between pairs of parallel cations, packing in a head-to-tail, offset π-stacking fashion, with a centroid-to-centroid distance of 3.6347 (12) Å. Neighbouring parallel cation pairs pack with aromatic planes at an angle of 57 °. The ammonium groups of pairs of parallel cations point to pairs of nitrate anions, interacting through strong, charge assisted N^+^—H···O^-^ hydrogen bonds, listed in Table 1. Each ammonium group is hydrogen bonded to three different nitrate anions through three bifurcated hydrogen bonds to six different oxygen atoms, as illustrated in Fig. 2(b). In each bifurcated hydrogen bond, one of the interactions displays an N^+^—H···O^-^ interaction angle closer to 180 °, while the angle of the second interaction deviates significantly more from linearity. In addition, for each bifurcated interaction, the two N^+^—H···O^- ^angles and the ^-^O—HO^-^ angle add up to approximately 360°. Each nitrate anion accepts three bifurcated hydrogen bonds from three different ammonium groups. The oxygen atom, O3, which accepts two approximately linear hydrogen bonds, exhibits a shorter N—O bond distance compared to the other N—O bonds.

The hydrogen bonding interactions result in a two-dimensional hydrogen bonded sheet, parallel to the *ab*-plane, as illustrated in Fig. 2(b). Two types of hydrogen bonded rings are present in the sheet. The larger of the two can be described by the graph set notation R^3^_6_(12), while the smaller ring is described by R^2^_1_(4) (Bernstein *et al.*,  1995).

### Experimental

3-Methylanilinium nitrate was prepared by the dropwise addition of excess concentrated nitric acid (0.90 ml, 70%, Saarchem) to a solution of *m*-toluidine (0.50 ml, 99%, Aldrich) in 20 ml chloroform (99%, Saarchem). Slow evaporation of the chloroform solution at room temperature gave colourless crystals.

### Refinement

All H atoms were refined using a riding model (HFIX 33 for N1 and C7), with C—H distances either 0.93 or 0.96 Å and N—H distances of 0.89 Å, and *U*_iso_(H) = 1.5*U*_eq_(C) or 1.2*U*_eq_(C) or 1.2*U*_eq_(N). The highest residual peak was 0.95 Å from atom H7B.

### Crystal data

**Table:**

C_7_H_10_N^+^·NO_3_^−^	*F*(000) = 720
*M**_r_* = 170.17	*D*_x_ = 1.322 Mg m^−^^3^
Orthorhombic, *P**b**c**a*	Mo *K*α radiation, λ = 0.71073 Å
Hall symbol: -P 2ac 2ab	Cell parameters from 3320 reflections
*a* = 10.6599 (14) Å	θ = 2.5–26.0°
*b* = 9.7800 (13) Å	µ = 0.11 mm^−^^1^
*c* = 16.401 (2) Å	*T* = 293 K
*V* = 1709.9 (4) Å^3^	Plate, colourless
*Z* = 8	0.40 × 0.32 × 0.05 mm

### Data collection

**Table:**

Bruker (Siemens) P4 diffractometer	1659 independent reflections
Radiation source: fine-focus sealed tube	1211 reflections with *I* > 2σ(*I*)
graphite	*R*_int_ = 0.032
φ and ω scans	θ_max_ = 26.5°, θ_min_ = 2.5°
Absorption correction: multi-scan (*SADABS*; Bruker, 2001)	*h* = −13→7
*T*_min_ = 0.968, *T*_max_ = 0.988	*k* = −9→11
8520 measured reflections	*l* = −18→20

### Refinement

**Table:**

Refinement on *F*^2^	Primary atom site location: structure-invariant direct methods
Least-squares matrix: full	Secondary atom site location: difference Fourier map
*R*[*F*^2^ > 2σ(*F*^2^)] = 0.040	Hydrogen site location: inferred from neighbouring sites
*wR*(*F*^2^) = 0.135	H-atom parameters constrained
*S* = 1.06	*w* = 1/[σ^2^(*F*_o_^2^) + (0.0693*P*)^2^ + 0.2901*P*] where *P* = (*F*_o_^2^ + 2*F*_c_^2^)/3
1659 reflections	(Δ/σ)_max_ < 0.001
111 parameters	Δρ_max_ = 0.17 e Å^−^^3^
0 restraints	Δρ_min_ = −0.14 e Å^−^^3^

### Special details

**Table:**

Geometry. All esds (except the esd in the dihedral angle between two l.s. planes) are estimated using the full covariance matrix. The cell esds are taken into account individually in the estimation of esds in distances, angles and torsion angles; correlations between esds in cell parameters are only used when they are defined by crystal symmetry. An approximate (isotropic) treatment of cell esds is used for estimating esds involving l.s. planes.
Refinement. Refinement of *F*^2^ against ALL reflections. The weighted *R*-factor wR and goodness of fit *S* are based on *F*^2^, conventional *R*-factors *R* are based on *F*, with *F* set to zero for negative *F*^2^. The threshold expression of *F*^2^ > σ(*F*^2^) is used only for calculating *R*-factors(gt) etc. and is not relevant to the choice of reflections for refinement. *R*-factors based on *F*^2^ are statistically about twice as large as those based on *F*, and *R*- factors based on ALL data will be even larger.

### Fractional atomic coordinates and isotropic or equivalent isotropic displacement parameters (Å^2^)

**Table:**

	*x*	*y*	*z*	*U*_iso_*/*U*_eq_	
N2	0.86066 (14)	0.20637 (15)	0.24233 (8)	0.0590 (4)	
C4	0.62873 (17)	0.1314 (2)	0.08553 (12)	0.0704 (5)	
H4	0.6324	0.1346	0.1421	0.084*	
O2	0.95202 (13)	0.13478 (15)	0.22538 (9)	0.0824 (5)	
C2	0.67695 (17)	0.01475 (19)	−0.03889 (11)	0.0659 (5)	
H2	0.7116	−0.0593	−0.0665	0.079*	
C6	0.57062 (16)	0.23133 (16)	−0.04117 (10)	0.0573 (4)	
H6	0.5344	0.3018	−0.0711	0.069*	
C5	0.57263 (16)	0.23767 (18)	0.04363 (11)	0.0619 (5)	
C3	0.67905 (19)	0.0215 (2)	0.04542 (12)	0.0774 (6)	
H3	0.7150	−0.0494	0.0751	0.093*	
C7	0.5124 (3)	0.3551 (2)	0.08774 (13)	0.0914 (7)	
H7A	0.4233	0.3413	0.0899	0.137*	
H7B	0.5303	0.4387	0.0594	0.137*	
H7C	0.5452	0.3603	0.1422	0.137*	
O3	0.77282 (13)	0.15739 (14)	0.28406 (8)	0.0733 (4)	
O1	0.85544 (14)	0.32743 (14)	0.22016 (8)	0.0765 (4)	
N1	0.61546 (14)	0.11602 (14)	−0.17012 (8)	0.0608 (4)	
H1A	0.5376	0.1350	−0.1862	0.091*	
H1B	0.6366	0.0328	−0.1872	0.091*	
H1C	0.6681	0.1772	−0.1911	0.091*	
C1	0.62205 (14)	0.12107 (16)	−0.08055 (10)	0.0516 (4)	

### Atomic displacement parameters (Å^2^)

**Table:**

	*U*^11^	*U*^22^	*U*^33^	*U*^12^	*U*^13^	*U*^23^
N2	0.0751 (10)	0.0567 (8)	0.0453 (7)	−0.0010 (7)	−0.0051 (7)	0.0024 (6)
C4	0.0682 (12)	0.0909 (14)	0.0520 (10)	−0.0142 (10)	−0.0053 (8)	0.0093 (9)
O2	0.0812 (10)	0.0760 (9)	0.0899 (11)	0.0165 (7)	0.0129 (7)	0.0099 (7)
C2	0.0653 (11)	0.0612 (10)	0.0712 (11)	0.0056 (8)	−0.0008 (9)	0.0071 (9)
C6	0.0678 (10)	0.0511 (9)	0.0531 (10)	−0.0049 (8)	0.0013 (7)	0.0044 (7)
C5	0.0663 (11)	0.0669 (11)	0.0524 (10)	−0.0152 (9)	0.0062 (8)	−0.0010 (8)
C3	0.0746 (12)	0.0840 (14)	0.0735 (12)	0.0043 (10)	−0.0102 (10)	0.0241 (11)
C7	0.1199 (18)	0.0888 (14)	0.0655 (12)	−0.0031 (13)	0.0233 (12)	−0.0117 (11)
O3	0.0808 (9)	0.0699 (8)	0.0692 (8)	−0.0048 (7)	0.0138 (7)	0.0074 (6)
O1	0.0983 (10)	0.0568 (8)	0.0746 (9)	0.0046 (7)	0.0019 (7)	0.0138 (6)
N1	0.0742 (10)	0.0545 (8)	0.0537 (8)	−0.0009 (7)	0.0008 (7)	−0.0042 (6)
C1	0.0550 (9)	0.0499 (9)	0.0501 (9)	−0.0075 (7)	−0.0011 (7)	0.0005 (7)

### Geometric parameters (Å, °)

**Table:**

N2—O2	1.2312 (19)	C6—H6	0.9300
N2—O1	1.2398 (19)	C5—C7	1.501 (3)
N2—O3	1.2550 (18)	C3—H3	0.9300
C4—C3	1.370 (3)	C7—H7A	0.9600
C4—C5	1.382 (3)	C7—H7B	0.9600
C4—H4	0.9300	C7—H7C	0.9600
C2—C1	1.375 (2)	N1—C1	1.472 (2)
C2—C3	1.385 (3)	N1—H1A	0.8900
C2—H2	0.9300	N1—H1B	0.8900
C6—C1	1.371 (2)	N1—H1C	0.8900
C6—C5	1.392 (2)		
			
O2—N2—O1	120.82 (16)	C2—C3—H3	119.7
O2—N2—O3	119.76 (15)	C5—C7—H7A	109.5
O1—N2—O3	119.40 (16)	C5—C7—H7B	109.5
C3—C4—C5	121.40 (18)	H7A—C7—H7B	109.5
C3—C4—H4	119.3	C5—C7—H7C	109.5
C5—C4—H4	119.3	H7A—C7—H7C	109.5
C1—C2—C3	117.87 (17)	H7B—C7—H7C	109.5
C1—C2—H2	121.1	C1—N1—H1A	109.5
C3—C2—H2	121.1	C1—N1—H1B	109.5
C1—C6—C5	119.96 (16)	H1A—N1—H1B	109.5
C1—C6—H6	120.0	C1—N1—H1C	109.5
C5—C6—H6	120.0	H1A—N1—H1C	109.5
C4—C5—C6	118.03 (17)	H1B—N1—H1C	109.5
C4—C5—C7	121.37 (18)	C6—C1—C2	122.05 (16)
C6—C5—C7	120.59 (17)	C6—C1—N1	118.52 (14)
C4—C3—C2	120.68 (18)	C2—C1—N1	119.41 (15)
C4—C3—H3	119.7		
			
C3—C4—C5—C6	−1.2 (3)	C1—C2—C3—C4	−0.5 (3)
C3—C4—C5—C7	177.24 (19)	C5—C6—C1—C2	−0.2 (2)
C1—C6—C5—C4	0.8 (2)	C5—C6—C1—N1	178.47 (14)
C1—C6—C5—C7	−177.68 (17)	C3—C2—C1—C6	0.1 (3)
C5—C4—C3—C2	1.1 (3)	C3—C2—C1—N1	−178.60 (16)

### Hydrogen-bond geometry (Å, °)

**Table:**

*D*—H···*A*	*D*—H	H···*A*	*D*···*A*	*D*—H···*A*
N1—H1A···O1^i^	0.89	2.05	2.943 (2)	178
N1—H1A···O2^i^	0.89	2.51	3.130 (2)	127
N1—H1B···O3^ii^	0.89	2.15	3.0221 (19)	167
N1—H1B···O2^ii^	0.89	2.37	3.078 (2)	136
N1—H1C···O3^iii^	0.89	2.01	2.879 (2)	166
N1—H1C···O1^iii^	0.89	2.47	3.176 (2)	137

Symmetry codes: (i) *x*−1/2, −*y*+1/2, −*z*; (ii) −*x*+3/2, −*y*, *z*−1/2; (iii) *x*, −*y*+1/2, *z*−1/2.
